# Beyond the labels: Classifying countries by child health outcomes – A cluster analysis of child mortality and child-health data

**DOI:** 10.1080/16549716.2025.2526315

**Published:** 2025-07-22

**Authors:** Edward Purssell, Sharron Frood, Rohit Sagoo

**Affiliations:** a Children’s Nursing, Anglia Ruskin University, Chelmsford, UK; b Child Health, City St. George’s University of London, London, UK; c Florence Nightingale Faculty of Nursing, Midwifery and Palliative Care, FACT Study Kings College London, London, UK

**Keywords:** Classifying countries, child health outcomes, cluster analysis, child mortality, child health data

## Abstract

**Background:**

Most health service classification systems are based on organisational components such as service provision, financing, and regulation. This study considers health systems using data focusing on child health outcomes, service provision, and selected social characteristics. This more accurately reflects the reality of health service provision for children, young people, and their families.

**Objective:**

To classify health systems based on child health data through cluster analysis and exploratory and descriptive data analysis.

**Method:**

Data were extracted from the current version of the UNICEF (2023) State of the World’s Children full dataset, concentrating on outcomes related to mortality. Cluster analyses were conducted, and a heatmap was produced to identify patterns and groups among countries and child health indicators. Row and column distances were calculated using the Euclidean distance, and clustering was performed using the complete linkage method. Each variable was centred and scaled using the scale command, allowing variables measured on different scales to be compared without those with large values being weighted more heavily. Countries that performed better or were less healthy than expected were identified through linear regression analysis using the ggplot2 package.

**Results:**

Analysis of countries by cluster reveals six main groups, characterised by child and maternal mortality rates, vaccination levels, access to maternal and child healthcare, access to water and sanitation, and population migration levels.

**Conclusion:**

Identifying patterns in outcomes and identifying countries that perform above or below expectations concerning child health can inform a more nuanced approach to improving a country’s child health outcomes.

## Paper Context

**Main findings:** Understanding child health and social care provision patterns and related outcomes is complex. Studies such as this may indicate further enquiry for identifying combinations of interventions that maximise health benefits.

**Added knowledge:** This study demonstrates that understanding the patterns of health provision and child health outcomes is instructive in developing policies related to child health.

**Global health impact for policy and action:** Policymakers and leaders within Health Systems must understand the reasons behind countries that have better-than-expected child health outcomes compared to current health expenditures. What are the nuances within countries that cause these patterns?

## Background

### Why is understanding child health outcomes (CHOs) important?

The United Nations Convention on the Rights of the Child commits countries to making the child’s best interests the primary consideration in all actions concerning children [1]. However, it is recognised that how this is operationalised will differ according to the availability of resources, local priorities, and other contextual factors, some of which may be independent of a country’s wealth or level of health spending. Furthermore, although the relative over- or underperformance of some health systems is well-documented, the successes or challenges of many others remain less well-known.

Despite the importance of local factors, it is still possible for countries to learn from the experience of others about the interventions or combinations of interventions that are most effective for promoting child health. The International Council of Nurses recognises the critical role that nurses should play in developing and maintaining high-performing, safe, appropriate, and sustainable primary care [2]. This should be based on delivering that care and understanding the effect of individual and bundles of interventions on important child health outcomes.

Disparities in health outcomes and the psychosocial factors contributing to them are evident early in life and persist throughout a person’s lifetime [3]. There are several methods for assessing the effectiveness of health systems. Modern guidance and decision-making methods emphasise the importance of focusing on outcomes that are the most important to care recipients [4]. These variations in provision and outcomes provide an almost limitless combination of factors to consider when evaluating the effectiveness of health and social care provision to children. However, understanding the patterns within this complexity may help us learn from and share best practices – for example, the Sure Start programme in the UK [5].

Patterns of illness, policy responses, and a country’s willingness and ability to fund and provide care change over time. Due to a rise in childhood obesity levels globally, the WHO has recently developed guidelines related to physical activity and sedentary behaviour, as well as policies regarding a healthy diet [6]. The initial WHO conference in 1978 was centred around what constitutes primary healthcare and focused on peace, shelter, and education [7]. Global health policy has had a longstanding shift from communicable to non-communicable diseases affecting populations [8]. However, communicable diseases continue to account for a substantial proportion of childhood morbidity and mortality.

Classifying healthcare systems can help in understanding the methods and patterns of healthcare provision between countries and across global regions. Furthermore, it may be possible to develop an understanding of features that can be generalised beyond the unique circumstances of any given healthcare system, providing insights that may be valuable in developing healthcare policy [9]. International comparisons of child health outcomes may also help identify the strengths and weaknesses of delivering care to children in different countries, allowing the sharing of best practices to improve child health outcomes.

Currently, most health service classification systems are based on organisational identifiers such as service provision, financing as a percentage of GDP, and health service regulation. This includes countries in both the Global South and the North. However, a modern systematic review by de Carvalho found that healthcare guideline methods emphasise the importance of concentrating on outcomes of tremendous significance for patients and other end-users [10]. While the organisation and financing of healthcare are essential, clinical outcomes are of more immediate relevance to patients and carers. Comparisons such as those undertaken by the Commonwealth Fund are limited to a few high-income health systems. However, these studies demonstrate no direct relationship between health service characteristics, such as access to care, care processes, administrative efficiency, equity, and healthcare outcomes [11].

In addition to being provision-focused, it has been noted that many classifications are centred on systems and processes from the perspective of those in the ‘Global North’ [10,12]. However, naive dichotomous categorisation of countries into the ‘Global North’ and ‘Global South’ or other broad categories may lead to assumptions about the quality of care and outcomes based on geography or other features, which do not accurately reflect the experiences of children and families. A significant amount of innovative practice and high-quality care is likely being provided in low- and middle-income countries, which are traditionally considered disadvantaged and have limited financial resources. Just as nursing models and definitions emphasise the importance of individualised care, countries, and systems should be evaluated on individual health outcomes rather than group-level labels that may not reflect the reality for individual children and families. Much innovation is happening outside of countries that are often studied and reported, and it is essential to highlight these developments to facilitate the sharing of best practices [13].

The ultimate goal of every Child Health System is to achieve the health outcomes that children and their families experience. Traditional approaches have categorised countries into broad categories such as the Global North and the Global South. We sought to develop a more nuanced approach based on CHOs. CHOs refer to the changes in health status in children that result from specific interventions, treatments, or health policies. They are crucial metrics in the fields of healthcare and public health, providing insight into the effectiveness of medical practices and health initiatives [14]. By analysing health outcomes, researchers and healthcare professionals can assess the quality of care provided and identify areas for improvement. The CHOs selected for this paper are pragmatic and exploratory.

This study, therefore, considers health systems in a way that differs from most similar analyses in two ways: it is outcome-focused on mortality and other child-health-related outcomes, and it evaluates and categorises countries based on the characteristics of their child-health provision and selected social characteristics. In line with the requirements of the UN Convention on the Rights of the Child [1], we also consider the influence of the availability of healthcare resources. Health systems have been defined as ‘all the activities whose primary purpose is to promote, restore, or maintain health’ [15].

## Aims and objectives

### Aim

This paper aims to compare and contrast child health systems, classifying them on the basis of selected characteristics of the country, service provision, and outcomes. This is accomplished using the principles of exploratory data analysis, which involves both descriptive and inferential data.

### Objectives

Identify key features of different health systems and patterns of child health provision and outcomes.

Using heatmaps and cluster analysis to visualise this.

To identify countries whose child health outcomes are above or below what might be expected based on health spending.

## Methods

### Research design

This descriptive and inferential study uses cluster analysis, regression, and visualisation. The general approach is consistent with the principles of Exploratory Data Analysis (EDA), and in particular that ‘the picture examining eye is the best finder we have of the wholly unanticipated’ [16]. To this end, we use visualisation of data in the form of heatmaps, cluster analysis to demonstrate relationships, and regression. Cluster analysis is an unsupervised machine learning technique that seeks to find similarities and differences between multiple variables in a dataset. It does so by identifying groups or ‘clusters’ of objects or responses, where those within a given cluster have greater similarities to each other than to those in different groups [17]. When applied alongside heatmaps, this both visualises the results and shows these relationships through a dendrogram. Crucially, there is no one ‘correct’ clustering result as there are a variety of methods to do this, making interpretation of results crucial.

### Sample, setting, and data collection

Data were extracted from the current version of the UNICEF State of the World’s Children full dataset, which concentrates on mortality outcomes [18]. The current health expenditure (CHE) per capita in US dollars was obtained from the World Health Organisation database [19]. Three countries (Holy See, Liechtenstein, and Tokelau) were not included in the dataset. Formal analyses were restricted to countries with a population of over 10,000,000. Detailed information on the variables entered into the model is provided in the Supplementary File.

### Ethical considerations

Data are in the public domain, and there are no specific ethical considerations regarding their use in this study. There are many broader issues regarding the ethics of data collection and the uses to which they are put, and we are cognoscente of the need to always act in the best interest of children and families.

### Data analysis and synthesis

Data were extracted, and each outcome variable was centred and scaled using the scale command, allowing variables which are measured on different scales to be compared without those with large values being weighted more heavily. Missing values were permitted but were excluded from the distance computations involving the rows or columns within which they occurred [20]. Other approaches to handling missing data, such as imputation, were not used because key assumptions, in particular that the data were missing completely at random or missing at random, could not be assumed [21]. We also considered the patterns of missingness to be important variables in the model. As an unsupervised machine learning technique, cluster results need both validation and interpretation. Thus, in line with the principles of EDA of visualisation and exploration, we looked not just at the branching level but also at what the underlying meanings might be. The cluster results were therefore integrated with other evidence and experience to explain the resultant patterns [22], particularly in answering the question, ‘What makes sense?’

Heatmaps and cluster analyses were undertaken using the pheatmap command in the R package pheatmap [23,24]. Cluster analysis is a two-step process. First, a matrix of distances between individuals is calculated, and this is then used to group individuals sequentially with those most similar. Most commonly, this is done using agglomerative methods, where each individual starts as their own cluster, while divisive methods start with one large cluster. Row and column distances were calculated using the Euclidean distance, and clustering was performed using the agglomerative average linkage method. There are no definitive rules regarding the choice of clustering methods. These were chosen because Euclidean distance is commonly used and is intuitive, while average linkage uses the average distance between points in the first and second clusters to link them together. This forms a middle-ground approach between nearest and furthest neighbour methods, takes account of cluster structure, and tends to provide robust solutions [25].

To identify countries with higher or lower neonatal and under-5 mortality outcomes than might be expected for their level of health expenditure, linear regression was conducted using the ggplot2 geom_smooth function to undertake linear regression with associated 95% confidence intervals [26].

## Results

**Figure 1.**
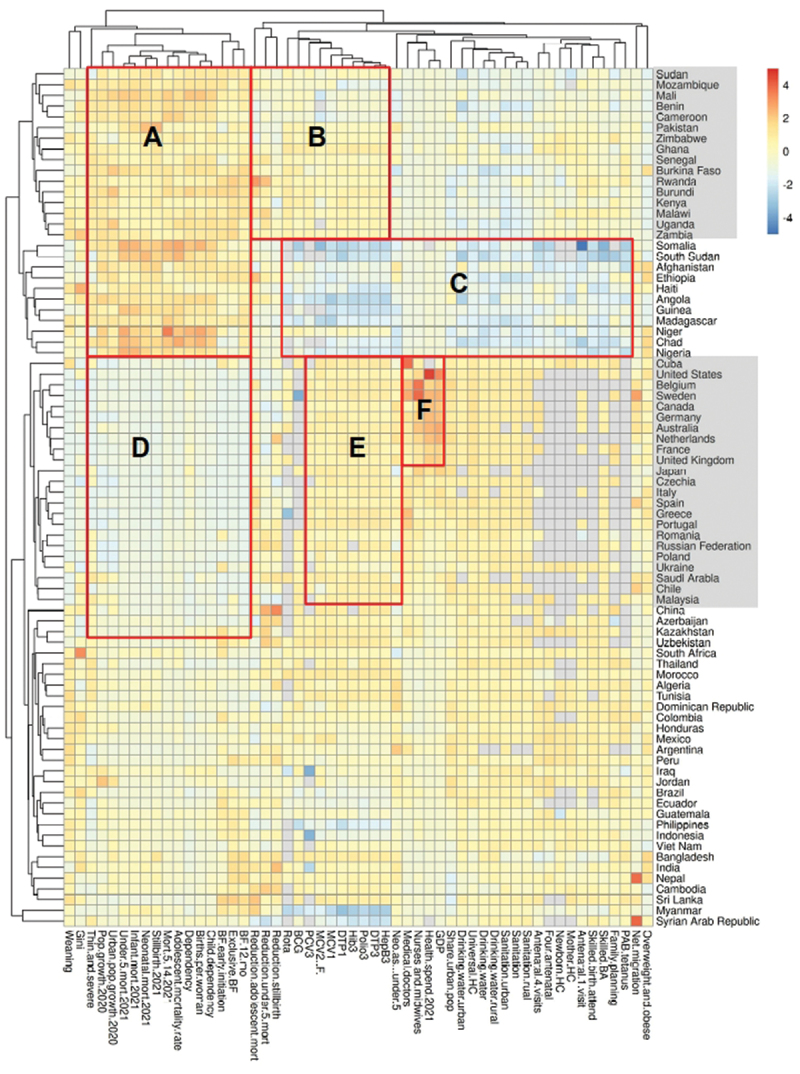
Heatmap of child-health-related outcomes and critical features of health services.

The heatmap and cluster analysis are shown in Figure 1. The colours indicate the standardised difference from the mean for each variable. Red and orange cells indicate above-average values, blue cells indicate below-average values, and grey cells indicate missing data within this cluster analysis. The red-orange cells in Cluster (A) denote countries with high rates of child and infant mortality. However, within this group, two patterns of child health provision are evident: those with low levels of vaccination, water, hygiene, and maternal and child health provision (E), and those with higher or variable levels. A large group of reasonably diverse countries have lower mortality (B), some of which have been able to commit higher resources (C), including Cuba and the United States. Greece and Portugal are noteworthy for having above-average numbers of medical doctors.

Analysis of countries by cluster reveals six main groups, designated as clusters A, B, C, D, E, and F. The variables entered into the analysis are presented in the Supplementary file.

Figure 1 illustrates the cluster analysis for countries with a population exceeding 10,000,000; the entire dataset is provided in the supplementary file. Six clusters of countries, A, B, C, D, E, and F, have been identified. What is immediately apparent is that the interpretation of these clusters does not reflect the simple division of the world into high-income, middle-income, and low-income geographies. The actual understanding of a child’s health outcomes is far more complex.

**Cluster A** comprises the countries listed vertically on the health map in Figure 1 below, namely Sudan and Nigeria. The child health outcomes that are most similar are those related to weaning, the Gini coefficient, population growth, urban population growth, under-five mortality rate, infant mortality rate, the number of stillbirths, the mortality rate of 5years to 14years, the number of births per woman, child dependency and early initiation of breastfeeding infants exclusively breastfed, and the reduction in adolescent mortality are all above average. This suggests that the identified outcomes are above average in these so-called low-income countries or countries in the Global South.

**Cluster B** comprises a group of countries, including Sudan, Mozambique, Mali, Benin, Cameroon, Pakistan, Zimbabwe, Ghana, Senegal, Burkina Faso, Rwanda, Burundi, Kenya, Malawi, Uganda, and Zambia, which are average in the following child health outcomes related to vaccination coverage. Rota Virus, BCGPCV3, MCV2, MCV1, DTP1, and Hep B 3. These are comparable to Cuba, the United States, Belgium, Sweden, Australia, the Netherlands, France, the United Kingdom, Japan, Canada, Italy, Spain, Greece, and Portugal.

**Cluster C** comprises Somalia, South Sudan, Afghanistan, Ethiopia, Haiti, Madagascar, Niger, Chad, and Nigeria, all located in the Global South. These present a primarily blue colour indicating lower than average levels in children being overweight and obese, net migration, PAB tetanus, Family Planning, Skilled birth attendants, Antenatal first visit Mother HC, four antenatal visits, rural sanitation, Sanitation, sanitation urban, drinking water rural, universal HC Share urban population, GDP Health spent in 2021, numbers of nurses and midwives, numbers of medical doctors, neonates to under-5 mortality rate. Regarding vaccinations, the rates for Hep B3, DTP3, Polio3, HB3, DTP1, MCV1, MCV2, PCV3, and BCG are all below the average rates of the countries included in the UNICEF 2023 data set.

**Cluster D**, which yields the same outcomes as Cluster A, is depicted in blue in Figure 1, indicating that the countries in this cluster are below the average. The countries in this cluster range from Cuba to Kazakhstan and include the UK, Australia, Poland, and Greece, and are identified as high- and middle-income countries located in the Global North.

**Cluster E** extends from Cuba to Uzbekistan. This cluster comprises the United States, Belgium, Sweden, Germany, the Netherlands, France, Italy, Spain, Greece, and Japan. These countries are located in the Global North and have average vaccination rates for the BCG vaccine and Hepatitis B.

**Cluster F** demonstrates that the identified countries, namely the United States, Belgium, Sweden, Canada, Germany, Australia, the Netherlands, France, the United Kingdom, and Japan, have above-average numbers of medical doctors, nurses, and midwives, spend above-average amounts of money on health, and the most significant proportion of GDP on health, and share urban populations are classified as high-income countries located in the Global North.

Examining the model variables shows the expected clustering of mortality, vaccination, sanitary and hygiene conditions, and resources. The Gini coefficient, as well as weaning, overweight, obesity, and net migration, showed the weakest relationship to other variables, as these were located in distinct clusters on their own.

There is a clear distinction in child mortality rates and child and maternal health interventions when comparing low-, middle-, and high-income countries across groups A to F. However, there are important exceptions. According to the World Health Organisation, maternal deaths occurred every 2 min worldwide in 2020, with nearly 95% of these deaths taking place in low to middle-income countries [27]. A study by Michel-Schuldt et al. found that midwifery-led care was a predominant feature in low-income countries, playing a key role in reducing maternal mortality and health disparities [12]. Midwives from low- to middle-income countries were deeply knowledgeable about the complexities, implementation, and effectiveness of maternal and child health interventions. They provided continuous care from birth through the first year of infancy. Child and maternal-focused interventions included education, sanitation, breastfeeding, infant nutrition, immunisation, and counselling. These interventions also led nurses and midwives to advocate for women’s empowerment, with a focus on improving child and maternal health outcomes. However, a lack of financial resources hindered access to health equity [28].

**Figure 2.**
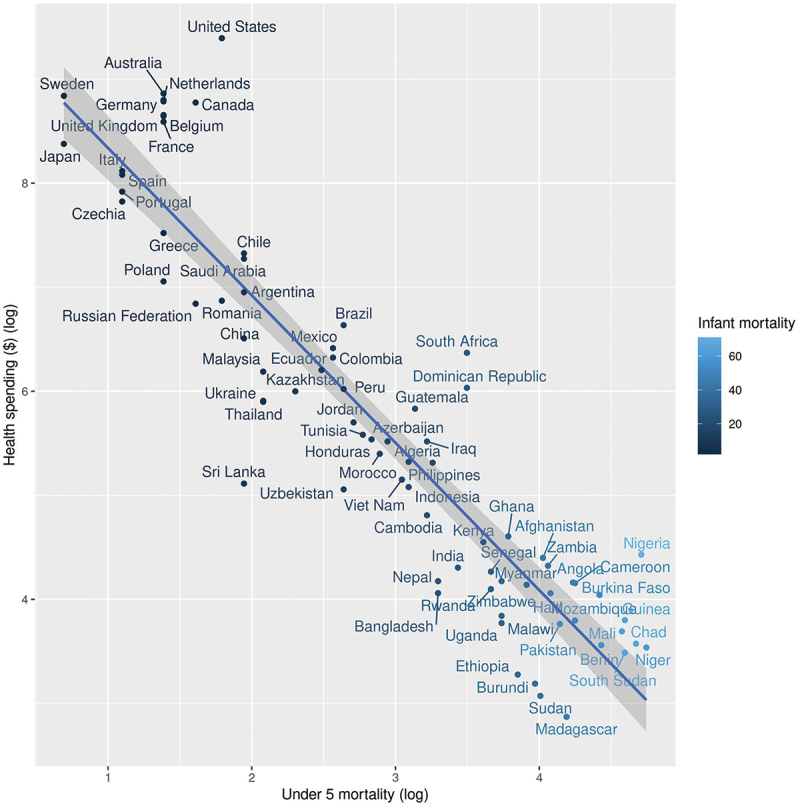
Scatterplot illustrating the relationship between Current Health Expenditure per capita in US dollars [CHE] and under-5 mortality.

This complexity is supported by the regression analysis of mortality rates under five against current health expenditure (CHE) per capita in US$, as shown in Figure 2. As might be expected, there is a negative relationship between health spending and under-5 mortality, with a regression coefficient of −0.59114 (95% CI: −0.6432154 to −0.5390623), and an adjusted R-squared value of 0.7512. This can be interpreted as the proportional change in mortality for each proportional change in health spending – for each 1% reduction in spending, there is a 0.59% increase in mortality [29]. Those countries above the regression line, such as the United States and Switzerland, have higher mortality rates than the model predicts for their level of health spending. In contrast, those below the line, including Belarus, Montenegro, and Sri Lanka, have lower mortality rates. Figure 1 demonstrates that the following countries spend above the average % of GDP on health: the USA, Belgium, Sweden, Canada, Australia, the Netherlands, France, the UK, and Japan.

Figure 2 helps illustrate patterns emerging between the current health expenditure (CHE) per capita in US dollars and under-5 mortality. This demonstrates the wide variation in both spending and outcomes. Countries with higher under-5 mortality rates than expected from a linear relationship include South Africa, the Dominican Republic, and several European and North American countries, most notably the United States. This latter cluster is most notable because all of these countries have very high levels of health-related expenditure.

What is apparent is that mortality rates for those under five are not dependent on the CHE. There are evident variations in these rates related to CHE per capita in US dollars and under-five mortality rates globally, which require further investigation to gain insight into the nuanced approaches within nations that determine these variables.

## Discussion

The approach of this study differs from many other attempts to categorise health services in that it is primarily outcome-based. The choice of the most appropriate child health outcomes to include in any such analysis is challenging to identify and is necessarily arbitrary. The child health outcomes identified in this paper are of two types: those that directly measure well-being, such as mortality and nutritional status, and those associated with future changes in well-being, such as vaccination. Additionally, it may be essential to go beyond traditional health-related measures, including education, transportation, and other social factors, to understand the landscape of child health fully. In presenting this discussion, consideration of the Sustainable Development Goals is essential. The insights presented in this paper could encourage further enquiry regarding countries with improved or above-average CHO, where the current health expenditure (CHE) per capita in US dollars is not proportional to these improvements.

The Sustainable Development Goals (SDGs) [30] and distal, intermediate, and proximal factors [31] are essential to consider in child health outcomes globally. The SDGs are goals that define specific targets relating to maternal and child mortality and universal health coverage. These targets are 3.2, 3.1, and 3.8, respectively. SDGs, also known as the Global Goals, were adopted by the United Nations in 2015 as a universal call to action to end poverty, protect the planet, and ensure that by 2030, all people will enjoy peace and prosperity. The 17 SDGs are integrated, recognising that action in one area will affect outcomes in others and that development must balance social, economic, and environmental sustainability. Sustainable Development Goal 3 relates to this paper as SDG 3 presents the following goal: to ensure healthy lives and promote well-being for all ages.

SDG 3.2 concerns Neonatal and child mortality. This goal set the target to end preventable deaths of newborns and children under 5 years of age by 2030, with all countries aiming to reduce neonatal mortality to at least 12 per 1000 live births and under-5 mortality to at least 25 per 1000 live births (SDGs) [30]. Regarding mortality rates, as presented in Section 3.1, maternal mortality is expected to be reduced by 2030, and the global maternal mortality ratio is projected to be less than 70 per 100,000 live births. Target 3.8 aims to achieve universal health coverage, encompassing financial risk protection, access to quality essential healthcare services, and safe, effective, quality, and affordable essential medicines and vaccines for all. These goals state what needs to be achieved. This paper presents valuable insights into nuanced approaches undertaken in countries related to maternal and child healthcare, where child health outcomes are not directly associated with a government’s GDP spent on health systems. For example, in Figure 2, the USA spends significantly more on healthcare (CHE) than Montenegro, yet Montenegro has a better under-5 mortality rate. Sapin and Italy have similar CHE, yet they have the same under-mortality rate as Belarus. However, all countries have the same under-5 mortality rate. This suggests that other factors may also contribute to preventing high mortality rates in addition to CHE. What is essential regarding the SDGs, however, is the intent within governments to meet these targets through their own and varied domestic policies related to child health. For example, the national plan for children in South Africa is for the period 2019–2024 [32]. This plan outlines the South African Government’s intention to implement policies to achieve the SDG targets for its children.

Child health combines specific and general interventions that can be viewed through multiple lenses. These include macro-, meso-, and microsystems, comprising distal, intermediate, and proximal features, as well as treatment and developmental approaches. The roots of this lie in Bronfenbrenner’s ecological systems theory, which provides an organising framework for influences on human development, with the proximal factors being the child and family [31]. Whilst this is a complex ecology to consider, it does provide a structure through which we can better understand some determinants affecting child health.

One way to understand the complexity of child health outcomes in countries is to consider the totality of child health provision, which encompasses a combination of specific and general interventions that can impact distal, intermediate, and proximal features, as well as treatment and developmental approaches. These are ‘distal’ to the child or ‘proximal’ [31]. The former are structural variables, including population structure and level measures; the latter refers to actual events experienced by the individual child [33]. This distinction is essential because health policymakers and individual practitioners have some control over the latter. For example, the Chronosystem [34] or distal factor is primarily influenced by government policy. The principle of best interests, as outlined in the UN Convention [1] and the Sustainable Development Goals (SDGs), would primarily impact government policies related to child health outcomes. The former are structural variables, including population structure and level measures; the latter refers to actual events experienced by the individual child in their home and school environment [33]. This distinction is essential because health policymakers and individual practitioners have some control over the latter, whereas control over structural factors is often more remote. Table 1 presents the details concerning proximal, intermediate, and distal factors.

**Table 1.** Distal, intermediate, and proximal factors.

Distal factors	Intermediate factors	Proximal factors
**Third-order macrosystem or Chronosystem** (cultural, policy and social systems)	**Second-order mesosystem** (operates through schools, community or neighbourhood services)	**First-order microsystem** (direct effect on the child and family)
Factors that influence care delivery at a level more than one step removed from the child	Factors one step removed from the child	Those that impact direct care delivery
Local or national vaccination policy *Provides direct and herd immunity*	Availability of vaccines in a clinic or local area *Provides direct and local herd immunity*	Ability to give vaccines to individual children Willingness of families to receive a vaccine *Provides direct immunity*
Policies regarding the provision of safe water and hygiene	Ability of local providers to deliver these	Use of these facilities

According to the WHO [15], global health policy has shifted from targeting diseases to targeting individuals [35]. Better access to essential health services, including vaccinations, oral rehydration therapy, and antibiotics for pneumonia, along with improvements in social conditions, such as higher standards of living and smaller families living on larger incomes, have been crucial factors in improving the survival rate of children [35]. Achieving positive health outcomes requires recognising that childhood mortality is influenced by more than just childhood interventions. The health of parents, siblings, grandparents, and other household members also plays a crucial role in a child’s well-being. Interventions during childhood can have lasting effects into adulthood. It is increasingly evident that health efforts in one generation can have a lasting impact on the next. For instance, ensuring proper nutrition for girls during childhood and adolescence can lower the risk of low-birth-weight babies, a key factor in early mortality [35].

To better understand health influences and structure them for effective health programmes, the World Bank and its partners have developed a life course approach [36]. This framework emphasises interventions across all life stages, with special attention to the reproductive period for women, including pregnancy and the start of a new generational cycle. Four key principles guide it:

1. Health interventions build on each other, meaning that earlier interventions influence later-life outcomes.
2. Sustaining health improvements at any stage requires interventions across multiple life stages.
3. Actions taken in one generation can have a lasting impact on the health of future generations.
4. Defining life cycle stages helps identify health risks for individuals and families.

Identifying significant health risks at each stage allows for targeted interventions, which can be implemented within the health sector or through broader strategies that influence household behaviours. Adopting a life cycle approach to reducing childhood mortality can foster collaboration across sectors, ensuring that resources are used more efficiently and effectively [36].

Any discussion on strategies to reduce childhood mortality must also consider the legal framework supporting these efforts. The Convention on the Rights of the Child, adopted by the United Nations General Assembly in 1989, explicitly upholds a child’s right to health and healthcare services. Article 24 of the convention mandates that signatory nations ‘pursue full implementation of this right and, in particular, take appropriate measures… to diminish infant and child mortality.’ Guidelines for implementing and monitoring the required actions have been developed and widely distributed to facilitate this [1].

The analysis of child health outcomes is critical because countries and outcomes can be compared through the pattern of responses across several variables, demonstrating the complexity of child health provision and its effect on mortality. Emerging ecobiodevelopmental frameworks suggest that nurses and other healthcare workers should go beyond traditional practice to adopt evidence-based strategies that promote educational achievement, increase economic productivity, and foster responsible citizenship, thereby maximising lifelong and intergenerational health [37]. This requires nurses to move beyond age- or discipline-based silos to view each stage of the life course as preparation for or the result of other developmental stages.

All those concerned with child health policy and delivery must actively develop, champion, and implement effective health and other related policies. As nurses are the largest group of healthcare professionals, they have a particular role in this work that is sometimes underestimated [38]. This broad overview of this type of research shows the variety of areas nurses might consider. However, considerable challenges include a lack of motivation and interest, inadequate education in this area, a shortage of mentorship, and limited time and resources. Nurses tend to be implementers rather than policymakers [39]. Although it is argued that nurses need to play a more significant role in policy development, some challenges remain in developing this role and the contribution that nurses can make needs to be identified [40]. One potential impediment is a greater need for statistical literacy [41]. It is arguable, for example, that greater involvement of nurses in policies related to COVID-19 might have shifted the emphasis of policy.

It is also essential to establish priority areas and interventions. Assigning a value to individual interventions and outcomes can be challenging, particularly when children are involved [42]. Key questions include which outcomes are most valued, what interventions provide the most value in achieving these, and how different combinations of interventions interact. Evaluation is complex, and it has been suggested that there are 12 possible elements. Four are currently widely used: quality-adjusted life-years (QUALYs), net costs (the balance of *incremental costs* and *benefit)*, changes in productivity, and any adherence improvement, while others are novel or less widely used: reduction in uncertainty (usually associated with diagnostic tests), fear or risk of contagion, insurance value, severity of disease, the value of hope where there is significant uncertainty, real option value where new treatments might become available in the future, equity, and ‘scientific spillovers’ resulting in new therapies [43]. The impact of the life course also needs to be considered; how does early exposure, for example, to malnutrition, affect people in later life?

In a more limited analysis, the OECD found six groups of countries that shared broadly similar healthcare institutions [44]. However, this was limited to a few high-income countries and focused on organisation and service payment. Here, we found a more complex structure where service features did not necessarily predict outcomes. This has also been demonstrated in comparisons of high-income countries, as assessed by The Commonwealth Fund report [11].

It has previously been noted that public health terminology contains many false dichotomies and outdated terms, including ‘Global North versus Global South’, ‘The Majority World versus Minority World’, and ‘Black Indigenous and People of Colour versus White’ and ‘global majority’ [45]. These data support their conclusion that we should use more nuanced terms, recognising the variations between countries shown here and areas within each country that were not assessed here. Furthermore, sweeping terms such as these fail to recognise the genuine advances that some countries have made and the lessons that others could learn from them. Countries with similar GDPs may exhibit different performance outcomes, and their performance across a range of indicators and outcomes is not necessarily uniform. Additionally, aggregated data, for example, under-5 mortality, can hide substantial variation in perinatal, neonatal, and death after these periods [46]. This analysis of health spending shows similar variability.

## Limitations

Although based on official data from the UNICEF and the WHO, the data quality may vary, and for some variables, there was a large amount of missing data. Some data on the official dashboards are also quite dated. However, these do represent the official data used by these and other organisations to inform policy, and the sources used are the seminal sources of information on this subject. As with any cross-sectional data, these also represent a snapshot of how things were at the point at which the data were collected. Furthermore, the choices of interventions were pragmatic, and we could not identify which interventions are likely to be of the highest value in preventing childhood mortality, which may also differ according to features such as underlying levels of child morbidity and mortality [47]. Although meta-analysis and randomised controlled trials represent the highest level of evidence, the data used here present real-world population-level data and come from organisations charged with creating international health policy.

We included the full range of vaccinations because each protects against a different infectious disease. In an inferential model, this would be problematic, while it may be overweight, vaccination readers can make their own choices regarding the importance of these interventions. Because these data are retrospective, essential changes may have occurred since their collection and publication. To this end, the pattern of responses, rather than individual countries’ positions, are crucial.

## Conclusions

These data suggest that patterns of child health and social care provision, as well as related outcomes, are complex and multifaceted. Studies such as this may indicate further enquiry for identifying combinations of interventions that maximise health benefits. Understanding the patterns of provision and outcomes is also instructive in developing policies related to child health and other areas of concern. All those in healthcare need to remember that the health of the child population will, in time, be reflected in the adults, making this a matter of general concern. However, despite numerous attempts to classify health systems and countries, this study has shown that beneath these broad groups, including those identified here, areas of concern and success require further investigation. Such an approach might enable the identification of new combinations of best practices to inform health policy.

## Supplementary Material

Supplementary_file_6th_June.docx

## Data Availability

The data supporting the findings of this study are available upon reasonable request from the corresponding author and are contained in the supplementary file submitted with this paper.
