# Supplementary material for: Beyond the labels: Classifying countries by child health outcomes – A cluster analysis of child mortality and child-health data
Source: Glob Health Action. 2025 Jul 22;18(1):2526315. doi: 10.1080/16549716.2025.2526315 (PMC12284984; doi:10.1080/16549716.2025.2526315)
Supplement: Supplementary_file_6th_June.docx [file ZGHA_A_2526315_SM7660.docx]

Supplementary file

Table 1 Terms of Reference for the Child Health Outcomes in Cluster Analysis.

| **Outcome Measure** | **Terms of Reference** |
| --- | --- |
| Weaning | Introduction of semi-solid food at 6-8 months |
| Gini | Presents the Gini Coefficient |
| Thin and Severe | Moderate and Severe underweight |
| Population Growth 2020 | Annual population growth in % |
| Urban Population Growth 2020 | Annual growth rate of urban populations % |
| Under 5 Mortality 2021 | Under 5 years mortality rate |
| Infant Mortality 2021 | Infant mortality rate |
| Neonatal Mortality 2021 | Neonatal mortality rate |
| Still Birth 2021 | Stillbirth rate |
| Mortality 5:14 2021 | Deaths among children aged 5-14 years |
| Adolescent Mortality Rate | Adolescent Mortality Rate 2021 |
| Dependency | The dependency ratio is an age-population ratio of those typically not in the labour force |
| Births Per Women | Total Fertility Rate (Number of Live births per woman) |
| Child Dependency | The child dependency ratio is a measure of the number of children population (ages 0-14) per 100 people of adults (ages 15-65). |
| BF Early Initiative | Early Initiation of Breast Feeding |
| Exclusive BF | Exclusive breastfeeding before 6 months of age |
| Breastfed at 12 months | Infants being breastfed at 12 months of age |
| Reduction in adolescent mortality | Annual reduction in the adolescent mortality rate 2000-2021. |
| Reduction in under-5 mortality | Annual rate of reduction in under-five mortality rates |
| Reduction in Still Births | The annual rate of reduction in the still-birth rate |
| Rota | Immunisation for preventable diseases Rota Virus Vaccine |
| BCG | Immunisation for preventable diseases BCG Vaccine |
| PCV3 | Immunisation for preventable diseases PCV3 Vaccine |
| MCV2 | Immunisation for preventable diseases MCV2 Vaccine |
| MCV1 | Immunisation for preventable diseases MCV1 Vaccine |
| DPT1 | Immunisation for preventable diseases DPT1 Vaccine |
| Hep B | Immunisation for preventable diseases Hep B Vaccine |
| Polio 3 | Immunisation for preventable diseases Polio 3 Vaccine |
| DTP3 | Immunisation for preventable diseases Polio 3 Vaccine |
| Hep B3 | Immunisation for preventable diseases Hep B 3 Vaccine |
| Neonatal Under 5 | Neonatal deaths as a percentage of under-five deaths |
| Medical Doctors | Physicians include generalists and specialist medical practitioners. Physicians (per 1,000 people) |
| Nurses and Midwives | Nurses and midwives include professional nurses, professional midwives, auxiliary nurses, auxiliary midwives, enrolled nurses, enrolled midwives and other associated personnel, such as dental nurses and primary care nurses. Nurses and midwives (per 1,000 people) |
| Health Spend 2021 | Current health expenditure (CHE) per capita in US$ |
| GDP | GDP per capita (Currently in US $) |
| Share of Urban Population | Refers to the percentage of a country's or region's total population residing in urban areas. |
| Drinking water urban | At least basic drinking water services |
| Universal Health Coverage | Service coverage sub-index |
| Drinking Water | At least basic drinking water services |
| Sanitation Urban | At least basic sanitation services |
| Sanitation | At least basic sanitation services |
| Sanitation Rural | At least basic sanitation services |
| Antenatal four visits | At least four antenatal visits |


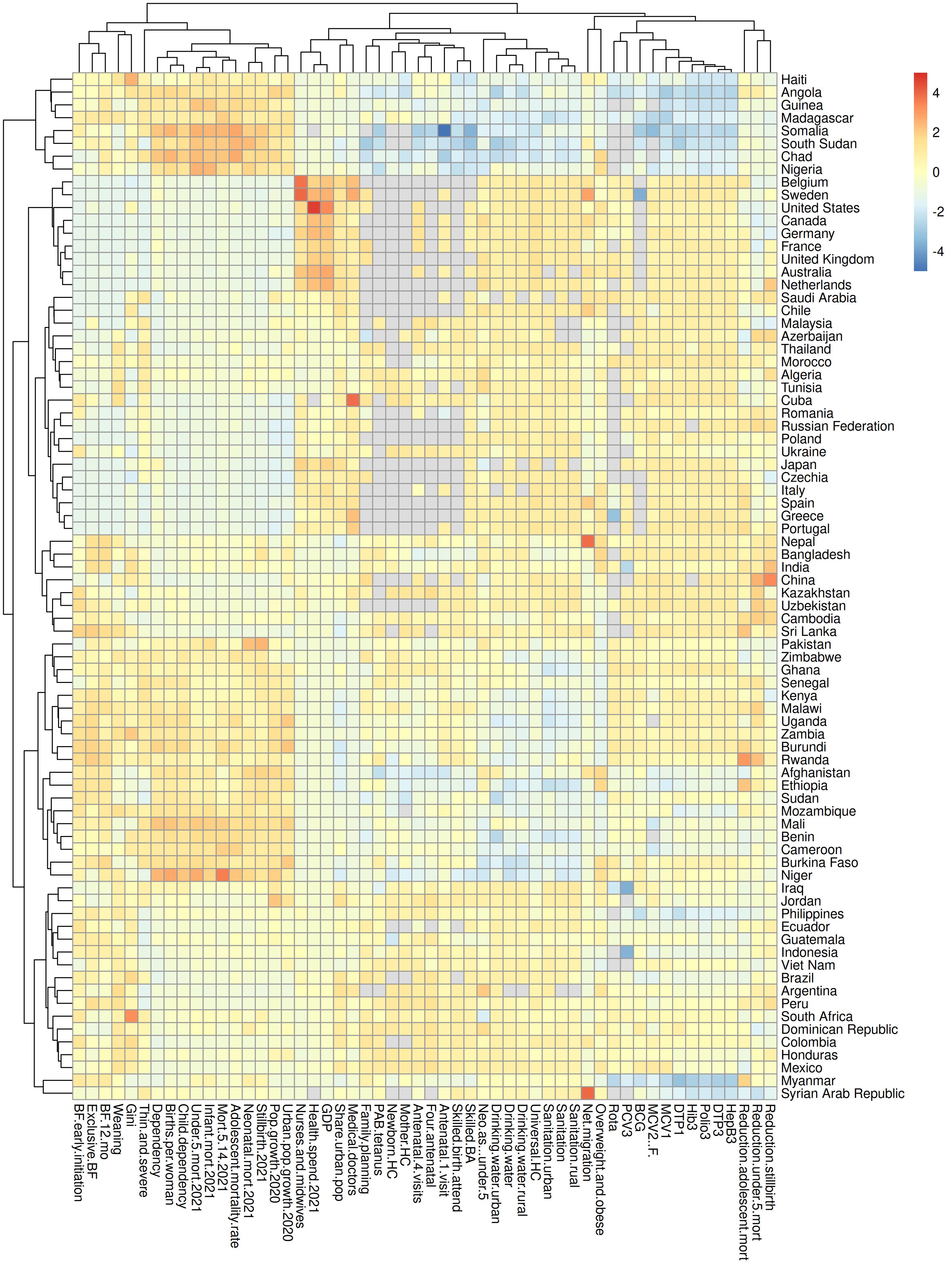
Figure 1 Euclidean – complete


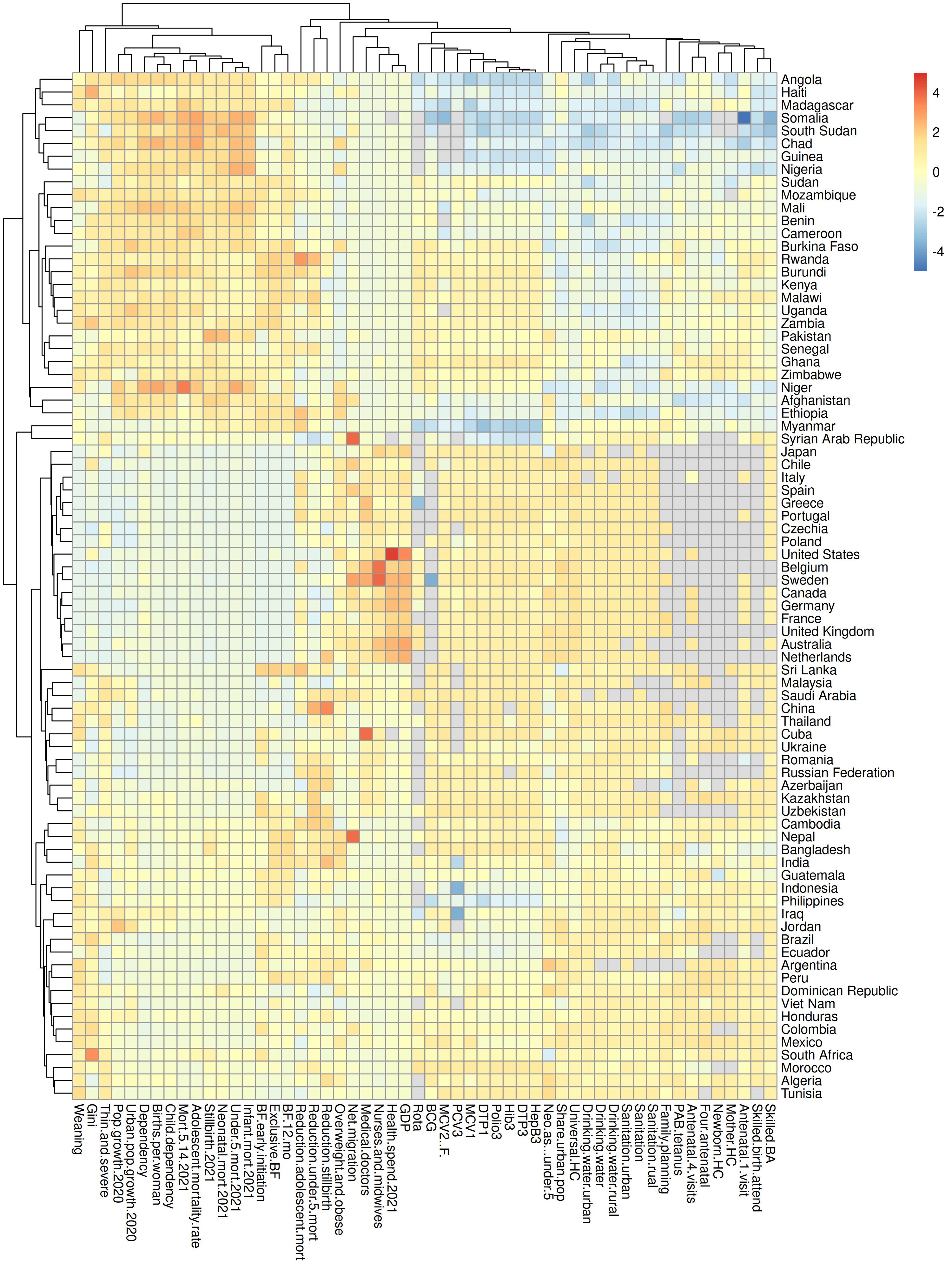
Figure 2: Manhattan -average


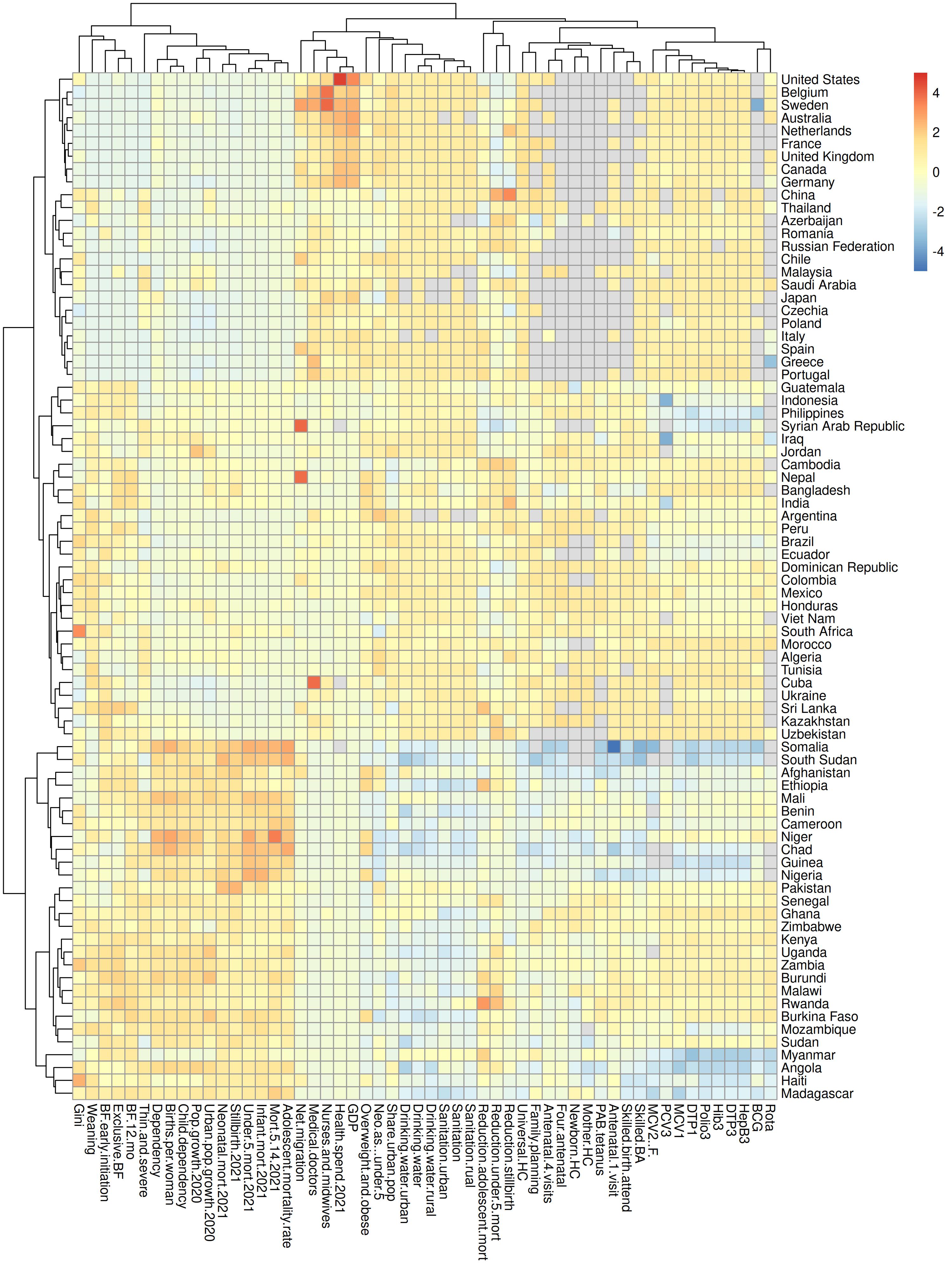
Figure 3: Manhattan - complete
